# Teaching Diversity in Public Health Through a Transformative Approach—An ASPHER Initiative

**DOI:** 10.3389/fpubh.2020.588111

**Published:** 2020-11-18

**Authors:** Lisa Wandschneider, Yudit Namer, Robert Otok, John Middleton, Oliver Razum

**Affiliations:** ^1^Department of Epidemiology and International Public Health, School of Public Health, Bielefeld University, Bielefeld, Germany; ^2^The Association of Schools of Public Health in the European Region (ASPHER), Brussels, Belgium

**Keywords:** diversity, public health education, capacity building, transformative learning, intersectionality, health equity

## Introduction

Diversity is increasingly taken into account in scientific research, funding schemes and publishing policies—as illustrated by the Frontiers statement on Diversity and Equity (1). Dimensions of diversity include (but are not limited to) gender identity, sexual orientation, migration background, ethnicity, socio-economic resources, age, physical capacity, religion, and/or region of residence. As hypothesized in minority stress theory and confirmed in empirical analyses, individuals categorized as members of a stigmatized minority population (e.g., LGBTQ people, foreign-born, refugees, and socio-economically deprived) experience a stressful social environment, and survival-related hypervigilance toward stressors (ranging from disregard to hate-motivated attacks) that are associated with higher rates of mental and physical health problems (2, 3). The Covid-19 pandemic is the latest example of how people experiencing marginalization, limited access to health and preventive services as well as precarious working and living conditions are at disproportionately higher risk of health-related and economic consequences (4).

## Diversity in Public Health Education—A Teaching Initiative

This recognition does not immediately translate to the teaching context. In the US, diversity is reflected in most medicine curricula and sometimes in public health schools (5). This is not yet the case in Europe where a systematic integration of diversity into public health curricula is urgently needed (6). Moreover, the notion of diversity is mostly restricted to “cultural competence” which reduces cultural diversity to ethnic difference (as a result of rising migration trends in immigration countries). Actually, diversity has more dimensions than ethnicity, and its dimensions often cumulate and interact, leading to what sociologists call intersectionality.

The Association of Schools of Public Health in the European Region (ASPHER) is an independent organization aiming to build the capacity of public health workforces for sustainable professionalization. In advocating for the improvement of public health education in Europe, ASPHER fully endorses the need of bringing diversity more into the focus of teaching. ASPHER therefore develops a policy on diversity competence to be an element of the ASPHER 2025 strategy. This approach goes beyond the notion of cultural diversity as ASPHER envisages an intersectional, holistic notion to reflect on multiple dimensions of diversity that affect the health of populations. To reach this goal, ASPHER member schools are developing teaching syllabi in a participatory process that offer guidance on how to address different dimensions of diversity that can add to existing curricula. The overall aim of the syllabi is to help establish a teaching program encouraging the reconsideration of normativities within public health while highlighting the intersectionalities between different markers of diversity. The teaching programme adopts a transformative learning approach to contribute to higher sensitivity toward diversity and simultaneously higher standards of the European public health education and training.

The theory of transformative learning is understood as the core principle of adult education and allows students to assess and if necessary, alter their perspectives about and responses to the world around them with the aim to educate socially responsible, autonomous actors (7). For learning to have a transformative quality, students need to (a) develop a critical awareness about the way they see the world, (b) revise their worldview to be more inclusive, and (c) transfer this worldview into practice (7). The teaching syllabi to be developed strengthen the teachers by offering them the tools to deem their teaching transformative, aiming for that transformation to spill over to future public health educators. Additionally, the impact of teachers is related to not only what they teach but which embodied perspectives they bring to the classroom. Faculty members with lived experiences of minority stress would reflect on previously overlooked viewpoints on health equity and social justice and facilitate more nuanced discussions. ASPHER thus encourages the diversification of the faculty members and student recruitment of public health schools to strive for a more profound transformation as examples of good corporate citizens in equality.

## Conclusions

The ASPHER teaching initiative focusing on diversity and health equity will help to prepare public health students to respond to the social challenges throughout their career. Besides intellectual and practical skills (such as qualitative and quantitative literacy, communication, teamwork, inquiry, and analysis) scholars should also be well-versed in social responsibility (7) for the complexity of health challenges within today's diverse society (8). Thus, the capacity to acknowledge and respond to the intersecting characteristics of diversity in a population increases the relevance and effectiveness of future public health programs for the society they address and thereby in reducing health inequalities. Mirroring the calls for standardization in the US (5) and by actively supporting the long-needed catch-up process in Europe, ASPHER can set new directions and higher standards of the public health education and training.

## Author Contributions

OR had the initial idea for the initiative and made significant contributions in the writing. LW and YN conceptualized the design of the initiative and drafted the initial manuscript. JM and RO revised the manuscript (earlier and final version) critically for intellectual content. All authors read and approved the final version of the manuscript.

## Conflict of Interest

JM is President of the Association of Schools of Public Health in the European Region (ASPHER). RO is ASPHER Director. OR is member of ASPHER's Executive Board. The remaining authors declare that the research was conducted in the absence of any commercial or financial relationships that could be construed as a potential conflict of interest.
